# Validation of a measurement instrument for parental child feeding in a low and middle-income country

**DOI:** 10.1186/s12966-018-0736-7

**Published:** 2018-11-20

**Authors:** Digna Niken Purwaningrum, Helda Yessy Maria Sibagariang, Jayashree Arcot, Hamam Hadi, Rasita Amelia Hasnawati, Risma Saski Rahmita, Rohan Jayasuriya

**Affiliations:** 1 School of Public Health and Community Medicine, Faculty of Medicine, The University of New South Wales, Level 3 Samuels Building, Sydney, 2052 Australia; 2 The Centre for Health Policy and Management, Faculty of Medicine, Public Health and Nursing, Universitas Gadjah Mada, Jalan Farmako Sekip Utara, Daerah Istimewa Yogyakarta, DIY 55281 Indonesia; 3 Pesawaran District Health Office Gedong Tataan, Pesawaran, Lampung 35371 Indonesia; 4 Food Science and Technology, School of Chemical Engineering, Faculty of Engineering, The University of New South Wales, Chemical Sciences Building F10, Sydney, 2052 Australia; 5 Universitas Alma Ata, Jalan Ringroad Barat Daya No.1, Tamantirto, Bantul, Daerah Istimewa Yogyakarta, DIY 55184 Indonesia

**Keywords:** Child feeding practices, Parental child feeding, Validation, Indonesia

## Abstract

**Background:**

Parental child feeding practices (PCFP) are a key factor influencing children’s dietary intake, especially in the preschool years when eating behavior is being established. Instruments to measure PCFP have been developed and validated in high-income countries with a high prevalence of childhood obesity. The aim of this study was to test the appropriateness, content, and construct validity of selected measures of PCFP in a low and middle-income country (LMIC) in which there is both undernutrition and obesity in children.

**Methods:**

An expert panel selected subscales and items from measures of PCFP that have been well-tested in high-income countries to measure both “coercive” and “structural” behaviors. Two sequential cross-sectional studies (Study 1, *n* = 154; Study 2, *n* = 238) were conducted in two provinces in Indonesia. Findings of the first study were used to refine subscales used in Study 2. An additional qualitative study tested content validity from the perspective of mothers (the intended respondents). Factorial validation and reliability were also tested. Convergent validity was tested with child nutritional status.

**Results:**

In Study 1, a confirmatory factor analysis (CFA) model with 11 factors provided good fit (RMSEA = 0.045; CFI = 0.95 and TLI = 0.95) after two subscales were removed. Reliability was good among seven of the subscales. Following a decision to take out an additional subscale, the instrument was tested for factorial validity (Study 2). A CFA model with 10 subscales provided good fit (RMSEA = 0.03; CFI = 0.92 and TLI = 0.90). The reliability of subscales was lower than in Study 1. Convergent validity with nutrition status was found with two subscales.

**Conclusions:**

The two studies provide evidence of acceptable psychometric properties for 10 subscales from tested instruments to measure PCFP in Indonesia. This provides the first evidence of the validity of these measures in a LMIC setting. Some shortcomings, such in the reliability of some subscales and further tests of predictive validity, require further investigation.

**Electronic supplementary material:**

The online version of this article (10.1186/s12966-018-0736-7) contains supplementary material, which is available to authorized users.

## Background

Parental care and practices, particularly feeding practices, are key factors influencing children’s dietary intake [1]. Parental child feeding practices (food parenting practices) are defined as the behaviors and strategies that parents use in a specific situation to manage the amount and type of food consumed by their children and the timing with which it is eaten [2, 3]. Through these practices, parents influence children’s taste, food preferences, eating habits, nutrition, and weight status [4]. Parents’ roles in relation to children’s food consumption include being providers, enforcers and role models, particularly during early childhood [5, 6]. The preschool years are a time in which children’s eating behaviors and food intake regulation are formed, and this remains stable in later childhood [7]. Mothers have the strongest influence on the preschool feeding experience and all aspects of food availability associated with children’s food intake [8]. Therefore, it is critical to target mothers of preschool children when seeking to understand associations between parental child feeding practices and child nutrition status.

Food parenting comprises a wide range of behaviors. Food parenting has been shown to influence children’s appetitive traits, and there has been investigation of various parental strategies, such as trying to control dietary intake by restriction, pressuring a child to eat, and monitoring what a child eats [9–12]. Restriction is applied to limit intake and prevent poor dietary behavior, particularly the consumption of food perceived to be unhealthy. Pressure is used to encourage children to eat more, and monitoring is applied to look after child eating behavior and regulate food intake [13]. In a recent review of food parenting, pressuring children to eat specific foods, restricting access to and eating specific foods, rewarding good behavior with food, and withholding food as punishment were classified as “coercive behaviors”. Keeping track of children’s consumption (particularly of unhealthy foods) and establishing meal time practices and environments (such as not allowing TV during meals) were identified as “structural” behaviors [14].

Over the last two decades, many instruments have been developed to measure aspects of food parenting. These developments were reviewed by de Lauzon-Guillain et al. [15], who recommended further evaluation for construct validity. A more recent review of the status of measures of food parenting made the observation that parental controls that allow children to make inappropriate eating decisions were not adequately captured in existing measurements, and that the structure of the food parenting environment is an important component of measurement [14]. In addition, the authors commented on the rapid growth of measures and stated that the field does not need more measures, but rather higher-quality measures with well-defined and operationalized constructs [14].

The Child Feeding Questionnaire (CFQ) has been one of the most widely used and tested measures of food parenting for the last 15 years [16]. It was originally developed in the United States to measure food parenting among parents with preschool children, but it has been extended to encompass parents of adolescents [16, 17]. The CFQ has been validated in most continents, but, with the exception of a single study in Vietnam [13], it has been validated exclusively in high-income countries (HIC). In general, most of these psychometric studies have confirmed the factorial validity of the CFQ’s subscales [18, 19]. However, there are inconsistent results, especially with the subscales addressing mothers’ perceptions (perceived parental and child weight), restriction, and pressure to eat [13, 20–22]. These discrepancies were mostly found in studies that included participants from different cultures and socioeconomic levels [13, 20, 22]. Many other instruments that purport to measure parenting practices introduce additional constructs and to varying extents overlap with the CFQ (see reviews by de Lauzon-Guillain et al. and Vaughn et al. [14, 15]).

The measures of parental perception in the CFQ have been tested for their relationships with measures of controlled feeding practices in most studies that have sought to validate parental feeding practice measures (see Additional file 1). There are differences depending on factors such as ethnic group and country. In their original study, Birch, et al. [9] found that measures of parental perceptions were positively correlated while pressure to eat was negatively correlated (*r* = − 0.26) with child weight (weight for height) [9]. Many studies have tested these hypotheses but the results are mixed (see Additional file 1).

In many low and middle-income countries (LMIC), growth faltering and stunting continue to be highly prevalent, and feeding strategies may differ from those used in high-income countries [23]. Many LMIC face a double burden of obesity and malnutrition, with many children undernourished. For example, in 2013 in Indonesia, an estimated 19.6% of children under five were underweight and 37.2% were stunted. At the same time, 11.9% were classified as overweight or obese [24]. However, there has not been an attempt to measure parental feeding practices in LMIC with validated measures. Such a study would logically seek to test whether measures validated in HIC could be generalized to such settings. The present study addresses this gap. It was conducted in Indonesia, a LMIC that has the fourth largest population in the world.

The aim of the research was to test the appropriateness, reliability, and validity of existing parental child feeding practice instruments, which were developed and tested in HICs, in a LMIC setting. In the first study, appropriate measures were selected, translated into Bahasa Indonesia, and then tested for factorial validity. In the second study, the results of Study 1 were used to refine these measures, which were then tested for content validity with mothers of children and for construct validity.

## Study 1: test of factorial validity of appropriate measures of parental child feeding practices

### Study setting

The first study was conducted in Lampung province in the southern part of Sumatra. Lampung province covers an area of over 34,000 km^2^ and had a population of 8.1 million in 2015 [25]. The Demographic Health Survey in 2012 found the median of completed school years among the female population (15–49 years old) in Lampung to be 8.3 years [26].

The study was conducted in one rural area in the Pesawaran district and two urban areas in the Bandar Lampung district. These three areas, covered by sub-district health centers (*Puskesmas*), were selected for feasibility of access and low socioeconomic levels.

### Selection of measures of parental child feeding practices

Two of the researchers (DP and HS) conducted a comprehensive review of literature to identify measurement instruments for parental child feeding practices. A panel of experts consisting of nutritionists, public health practitioners, a medical practitioner, a sociologist, and a linguistic specialist (all from Indonesia) was convened. They were tasked with selecting the most appropriate dimensions by scrutinizing the relevance of the items in each subscale to child feeding practice in Indonesia (in the instruments reviewed, these dimensions were measured by subscales of the instrument, for example “pressure to eat”). The panel identified that the Child Feeding Questionnaire, developed by Birch et al. [9] and the Comprehensive Feeding Practices Questionnaire (CFPQ), developed by Musher-Eizenman et al. [27], were most appropriate for the setting, and covered the widest aspects of the topic. The suitability of each of the subscales and items were discussed one by one for their content validity, based on the panel’s expertise and experience of parental child feeding practices in Indonesia. Thirteen subscales (52 items) were selected from the CFQ and CFPQ, including all seven subscales of the CFQ: perceived responsiveness, perceived parent weight, perceived child weight, child concern, monitoring, pressure to eat, and restriction (for health). Six of the 12 subscales of the CFPQ were selected: child control, emotional regulation, encourage balance and variety, environment, food as reward, and restriction for weight. Three subscales were common to both instruments. When there was overlap in subscales and items between the two instruments, it was decided, where possible, to retain a complete subscale from an instrument. For instance, the CPFPQ instrument was used for items about “food reward” as the CFQ contained only two of these items and they were located in another subscale (restriction).

The instrument was translated into Bahasa Indonesia by a bilingual researcher using standard translation procedures [28, 29]. The translations were submitted to the panel of experts who selected the subscales. They discussed contextual and cultural factors that may influence the understanding of each item, and whether the specific terms represent the intended meaning in English. Where there was agreement, the expert panel made changes to the translated instrument. A different bilingual person fluent in Bahasa and English translated the instrument back into English.

### Participant recruitment and data collection

The respondents were mothers (15 to 49 years) and their children aged between 24 and 59 months listed in Integrated Service Post (ISP or *Posyandu* in Indonesian) registers in one rural area (Pesawaran) and two urban areas (in Bandar Lampung). The youngest child in the family was selected in cases where a family had more than one child aged 24–59 months. The study excluded mothers who worked overseas or were out of town because the study required anthropometric measurement of the mother. Where a child was born pre-term (< 36 weeks), or the child had congenital and developmental abnormalities or chronic disease/allergy, as ascertained by the local health worker were excluded from the study.

For the study, community health workers (CHWs) responsible for maintaining *Posyandu* registers in the study areas prepared a list of potential respondents. Once potential respondents were identified, the CHWs were trained to select a sample of 10–15 children from their registers, using systematic sampling, and to contact the eligible participants. As there were some difficulties in recruiting participants in some areas, two *Posyandus* were asked to sample up to 20 children. After the list of eligible study participants was obtained from the ISP, eligible participants were invited by CHWs to visit the ISP. Researchers then confirmed the eligibility of the participants and obtained written consent. Trained enumerators administered the questionnaire, and measured the child and mother during the ISP visit. In total, 154 mothers and child dyads participated in the study. Data were collected in July and August 2015.

Three research assistants measured the weight and height of the mothers and children. Weight measurements were performed twice using a calibrated digital scale (CAMRY EB9003). Height was assessed twice using a calibrated portable stadiometer (Staturemeter MD16120003). The mean of the two measurements of weight and height were used to calculate the BMI of mother and child respectively. Children’s BMI *z* scores were determined using WHO Anthro version 3.2.2 following the of WHO’s recommended child growth standard [30]. We also categorized children’s nutritional status based on International Obesity Task Force (IOTF) BMI cut-offs [31]. The IOTF standard considers children’s sex, age, and BMI in determining children’s nutritional status. Mothers’ BMIs were calculated and categorized using the proposed cut-offs for Asian populations [32].

### Statistical analysis

Descriptive statistical analysis was carried out for the demographic characteristics and BMI of mothers and children. Confirmatory factor analysis was used to test the factorial validity of the thirteen-factor model. The response options were based on five item Likert scales, which had ordinal properties. As there was skewness and/or kurtosis in the data, the assumptions of multivariate normality were often violated. Best practice recommendations were followed to analyze the data using the mean and variance adjusted weighted least square (WLSMV) estimator available in mPLus software [33]. The CFA model testing procedures of model specification, identification, and estimation were followed to test model fit [34, 35]. Model fit was assessed using the following indices: root mean square error of approximation (RMSEA), ideally less than 0.06; Tucker Lewis Index (TLI); and Comparative Fit Index (CFI), ideally > 0.90 [34]. We judged the acceptability of model fit by the relative closeness of fit indices to the ideal values and re-specified the models based on both statistical (modification indices, factor weights) and substantive grounds. Instrument convergent validity was assessed by examining the association of the mean factor scores of each subscale with the child BMI *z* score adjusted for age and sex. Cronbach’s alphas were calculated to assess the internal reliability of the subscales. The STATA program and mPlus was used for these analyses.

### Results

Of the 154 participants in Study 1: 53.3% were from urban areas; 48% of the children were in the age group 22 to 36 months; 51.95% of the mothers were in the age group of 26 to 35 years; 53.25% of the mothers were overweight or obese; of the children, there were more girls (59.1%) than boys (40.9%); 26.6% of mothers were of an ethnicity other than Javanese; 35.7% of mothers had low educational attainment (≤ elementary school); most of the mothers identified as housewives (83.77%) (see Table 1).

**Table 1:** Basic characteristics of mothers and children from Studies 1 and 2

	Demographic variables	Mean (SD) or n (%)		*p*-value
		Lampung	Yogyakarta	
1	District areas			0.01**
	- Rural and suburban	72 (46.75%)^a^	202 (84.87%)	
	- Urban	82 (53.25%)	36 (15.13%)	
2	Children’s age (months)			0.05
	22–36 months	74 (48.05%)	92 (38.66%)	
	37–48 months	50 (32.47%)	75 (31.51%)	
	49–60 month	30 (19.48%)	71 (29.83%)	
3	Mothers’ age (years)			0.88
	16–25	25 (16.23%)	45 (18.91%)	
	26–35	80 (51.95%)	125 (52.52%)	
	36–45	47 (30.52%)	65 (27.31%)	
	46–55	2 (1.30%)	3 (1.26%)	
	> 55	0 (0%)	0 (0%)	
4	Mothers’ BMI (IOTF proposed cut-off for Asian adult population)			0.24
	< 18.5	17 (11.04%)	14 (5.88%)	
	18.5–22.9	55 (35.71%)	89 (37.39%)	
	≥ 23–24.9	23 (14.94%)	50 (21.01%)	
	25–29.9	43 (27.92%)	65 (27.31%)	
	≥ 30	16 (10.39%)	20 (8.40%)	
6	Children’s sex			0.16
	Boy	63 (40.91%)	127 (53.36%)	
	Girl	91 (59.09%)	111 (46.64%)	
7	Mothers’ ethnicity			0.01**
	Java	113 (73.38%)	236 (99.16%)	
	Non-Java	41 (26.62%)	2 (0.84%)	
8	Mothers’ education			0.01**
	≤ Elementary school	55 (35.71%)	20 (8.40%)	
	Graduated from junior high school	52 (33.71%)	78 (32.77%)	
	Graduated from senior high school	45 (29.22%)	117 (49.17%)	
	Graduated from college/ university	2 (1.30%)	22 (9.24%)	
	Others	0 (0%)	1 (0.42%)	
9	Mothers’ occupation			0.04**
	Housewife	129 (83.77%)	178 (74.79%)	
	Others	25 (16.23%)	60 (25.21%)	
10	Children’s nutritional status (Ministry of Health, Republic of Indonesia)			0.01**
	< −3 SD	12 (7.79%)	2 (0.84%)	
	- 3 SD to - 2 SD	13 (8.44%)	8 (3.36%)	
	- 2 SD to 2 SD	127 (82.47%)	216 (90.76%)	
	> 2 SD	2 (1.30%)	12 (5.04%)	
11	Children’s nutritional status (IOTF)			0.01**
	- Thinness 3	25 (16.23%)	9 (3.78%)	
	- Thinness 2	19 (12.34%)	19 (7.98%)	
	- Thinness 1	37 (24.03%)	62 (26.05%)	
	- Normal	69 (44.81%)	128 (53.78%)	
	- Overweight	2 (1.30%)	11 (4.62%)	
	- Obese	2 (1.30%)	5 (2.10%)	
	- Morbid	0 (0%)	4 (1.68%)	

**p < .05

^a^Study 1 did not cover a suburban area, percentage only from rural area

**Fig. 1:**
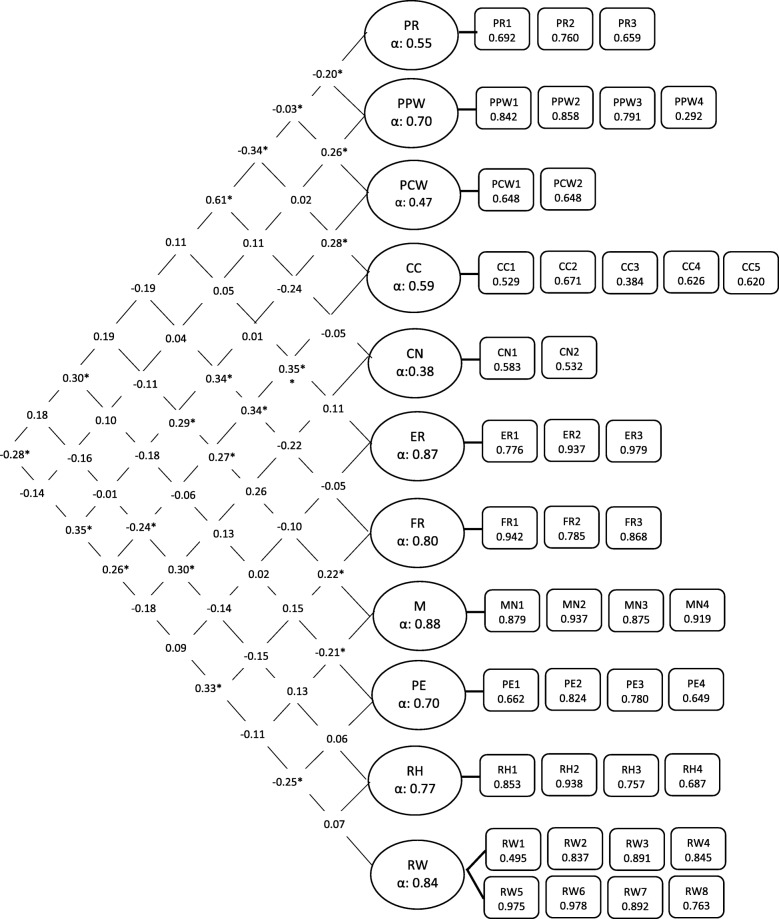
The Parental Child Feeding Practices Model in Study One. The PCFP model with standardized estimates fitted in a sample of Study 1, factor correlations and reliabilities (*n* = 154)

The analysis commenced with all 13 subscales and a series of iterations were conducted, based on accepted methods of identifying the best model to be retained, based on both statistical and substantive arguments. The first models included items from the two scales of CFPQ – “encourage balance and variety” and “environment” – which were found to have poor model fit [CFI 0.89, TLI 0.88, RMSEA 0.05 (90% CI: 0.049–0.060), WRMR 1.25]. There were also item inter-correlations with other subscales. Some of the questions asked were about food stored in the home and families in these settings did not store food but bought most of their food daily. Both of these subscales were removed on statistical and substantive grounds. We found very low loading of one item from the perceived child weight (PCW) scale. The item in question was PCW3 (“your child as a preschooler”) for which there were 111 missing values (72%). In this study 48% were under the pre-school age of 3 years and others may have misunderstood that children need to be enrolled in pre-school. In addition, a negative loading was seen with item CN3 (“concern about the child being overweight”) in the subscale “concern for child weight” (CN), which resulted in a non-positive definite matrix. These two items were taken out. The overall fit of the model with 11 factors was satisfactory based on accepted fit indices. The χ2/df = 1.31 (*P* < .01); RMSEA =0.045 (90% CI = 0.036–0.052); CFI = 0.95 and TLI = 0.95; with a WRMR 1.02. The factor correlations of these scales are shown in Fig. 1. The findings show that all items were meaningful indicators of the factors, with all of the loadings above 0.5, with a few exceptions: PPW4 did not load well (0.29) as given the culture mothers did not perceive themselves as “overweight” and the response was skewed. CC3 (0.38) was low and RW1 (0.495) marginal. Further iteration of models was not attempted, to allow retention of original subscales.

Discriminant validity of the scales was observed among the 11 scales as factor correlations were modest and in the correct direction with most in the range between 0.2 and 0.35 (see Fig. 1). Factor correlations between parental perceptions and feeding practices were examined. Perceived responsibility (PR) was positively related to concern about child weight (CN) (*r* = 0.61, *p* < .05) and pressure to eat (PE) (*r* = 0.30, *p* < .05), and negatively related to restriction for weight (RW) (*r* = − 0.28, *p* < .05). Perceived child weight (PCW) was positively related to child control (CC) (*r* = 0.28, *p* < .05), and monitoring (MN) (*r* = 0.29, *p* < .05) and restriction for weight (*r* = 0.35, *p* < .05). Restriction for health (RH) was the only factor significantly related to concern about child weight (*r* = 0.30, *p* < .05). Reliability, as measured by Cronbach’s alpha, was reasonable with values ≥0.70 except for PR, PCW, CC, and CN.

The tests of convergent validity between subscale scores and nutrition status did not show any significant associations (*p* > .05). A test for linear trends was also not significant.

### Discussion and plans for selection of measures for Study 2

The CFA provided good support for the factorial validity of the original Birch et al. [9] instrument in this setting. The food reward (FR) scale, which included two items from the Birch instrument, was included as a separate subscale (from the CFPQ) with three items. Two subscales, perceived child weight (PCW) and concern for child weight had only two items in the final model. Further investigation is needed on these measures. Of the six subscales included from the Musher-Eizenman instrument (CFPQ), the encourage balance and variety (EBV) scale was not included in the final CFA model. It was decided that, given that the setting and the nature of food storage was very different to that in the west (as most families purchased food or harvested food from their gardens daily), the scale on food environment did not portray the actual situation. There was also overwhelming evidence, both from the interviews that were conducted in Study 2 (see below) with mothers and the flooring effects on the item “concern for weight” found in the results of Study 1, that mothers were not concerned about the weight of the child, and they were not acting to restrict diet for weight control. Although restriction for weight showed factorial validity and good reliability, it was agreed to take this scale out for Study 2. In addition, taking out this lengthy subscale also reduced the respondent burden in Study 2.

## Study 2: test of construct validity of a refined instrument with a new sample

### Study setting

The second study was conducted in the Special Region of Yogyakarta province in the central part of Java. In 2015, the population was estimated at 3.7 million with a density of approximately 1155 per square kilometer [36]. A majority of female population (15–49 years old) had completed middle school, with a median 11.2 school years completed [26].

Data collection was conducted in three districts in the Special Region of Yogyakarta: rural areas in Bantul district, suburban areas in Sleman district, and urban areas in Yogyakarta Municipality. The study was undertaken from July to August 2016. The study sites were selected for their low socioeconomic areas and relatively high proportion of undernourished children.

### Methods of Study 2

As this study commenced approximately 6 months after the completion of Study 1, it was possible to incorporate the findings from Study 1 and refine the instrument used in Study 2. The following decisions were made:

i). Three measures (subscales) were removed on either substantive or statistical bases, as discussed above. Ten measures (subscales) were selected for the second study: perceived responsibility (PR, 3 items), perceived parental weight (PPW, 4 items), perceived child weight (PCW, 4 items), concern for child weight (CN, 3 items), child control (CC, 5 items), emotion regulation (ER, 3 items), food as reward (FR, 3 items), monitoring (M, 4 items), pressure to eat (PE, 4 items), and restriction for health (RH, 4 items).
ii). The item wording of the subscale perceived child weight, in which one item asked about the child’s weight as a preschooler, was found to create issues because many children were not yet preschoolers. This resulted in many missing values in the first study. The items were changed to reflect periods that were relevant (please see new wording in Additional file 2b).

### Qualitative study for content validation

A qualitative study was conducted using in-depth interviews with 24 mothers (aged 15 to 49 years) who had a child aged between 24 and 59 months. This study was conducted in March 2016. Mothers’ perceptions and experiences of their children’s eating behaviors were explored using open-ended questions. Interviews were conducted in Bahasa Indonesia by a researcher who was trained in qualitative research, and a note taker (research assistant). All interviews were audio recorded using a digital recorder and transcribed.

Methods previously used by Nilsson et al. [37] were followed in analyzing the data. The ten subscales used in Study 2 (derived from CFQ and CFPQ) were used as predetermined themes. Two researchers identified “pieces of text” that fitted each theme in each transcript. Then a match was made of whether they covered the intentions of each item. This is illustrated in Additional file 3. The two researchers made and compared separate analyses. Any discrepancies were discussed and consensus was achieved between the researchers. The results are given in Table 2. The inter-rater reliability, Cohen’s Kappa was 0.77 (*p* < .05).

**Table 2:** Content validity and inter-rater reliability

Subscale	Number of respondents who had statements that matched the subscale (Researcher A) N respondent = 24	Number of respondents who had statements that matched the subscale (Researcher B) N respondent = 24
Perceived Responsibility	23	24
Perceived Parental Weight	19	19
Perceived Child Weight	24	24
Concern about Child Weight	20	21
Food as Reward	21	21
Monitoring	15	15
Pressure to Eat	22	22
Restriction for Health	20	20
Child Control	24	24
Emotion Regulation	16	16

Cohen’s Kappa 0.77 (*p* < .05)

### Cognitive interviews

The aim of the cognitive interviews was to elicit from respondents similar to the study participants whether the instrument was comprehensible and clear [38]. Methods used previously by Knafl et al. [39] were used in our study. Five mothers with a minimum education level of senior high school completed this test.

We applied the verbal probing approach to ascertain respondents’ understanding and interpretation of each item. The cognitive interviews were conducted with individuals and a researcher interviewing each participant in person. The researcher read each item and the participants were asked what it meant to them, whether they experienced any problems in determining the meaning of the item, and what concerns participants had about the item’s wording. The researcher then asked whether the statement (question) was clear or unclear, easy or difficult to understand, whether there were offensive words, and the meaning of the statement to the respondent.

Results from the cognitive interviews were used to inform the improvement of items included in the validation study. We adjusted the wording order in PR3 and omitted a word in CC2. There was no difference in the meaning of these items when we translated them back into English.

### Participants in Study 2 – measurement validation survey

The participants in Study 2 were mothers aged between 15 and 59 years with children aged from 24 to 59 months who lived in one of three districts in the Special Region of Yogyakarta, namely Yogyakarta Municipality (urban), Sleman (suburban), and Bantul (rural). The inclusion and exclusion criteria used in Study 1 were followed. We purposively selected four integrated service posts in urban areas, seven ISPs in suburban areas and two ISPs in rural areas. After the list of eligible study participants was obtained from the ISP, eligible participants were invited by community health workers to visit the ISP. Researchers then confirmed the eligibility of the participants and obtained their written consent. The enumerators administered the questionnaires to the mothers during ISP visits. In total, 238 mothers participated in the survey. Data were collected in July and August 2016.

### Measures

Mothers’ demographic characteristics, such as age, highest education level, occupational status, ethnicity, and category of residential area, were collected. For the children, data collected included sex, age, and ethnicity. The PCFP questionnaire consisted of the 10 subscales listed above. None of the items were reverse coded. The PCFP used is provided in Additional file 2b.

The data collection protocols were the same as in the previous study.

### Statistical analysis

Following descriptive analyses, confirmatory factor analysis was used to test the factorial validity of the measures used. The same best practice recommendations were followed to analyze the data using the mean and variance adjusted weighted least square (WLSMV) estimator available in mPLus software [33]. The CFA model testing procedures of model specification, identification, estimation, and testing model fit were followed, as described in Study 1 [34, 35]***.*** Instrument convergent validity was assessed by examining the association of the mean factor scores of each subscale with child BMI *z* score adjusted for age and sex. ANOVA test for trend was conducted. Cronbach’s alphas were calculated to assess the internal reliability of the subscales. The STATA program and mPlus was used for these analyses.

### Results

The basic characteristics of the mothers and children who participated in the second study were as follows: most participants (84.9%) were from rural and suburban areas; 38.7% of children were in the age group 22 to 36 months; approximately 52.5% of the mothers were in the age group of 26 to 35 years; according to the proposed cut-off for Asian populations, the total percentage of mothers who were overweight or obese was 56.7%; 53.4% of children were male; as Study 2 was conducted in Java, 99% of mothers were Javanese; only 8.4% percent of mothers had less than elementary school education; 74.8% of mothers were housewives (see Table 1).

#### Item analysis

Study 2 item distribution, characteristics of each subscale, mean, standard deviation, skewness, and kurtosis are provided in Additional file 2b. All items in subscales perceived responsibility (PR), monitoring (MN), pressure to eat (PE), and restriction for health (RH) were negatively skewed. The other subscales had both positive and negative skewed items. Kurtosis ranged from 1.73 to 6.59. All of the items were within acceptable limits for normality, being < 2.0 for skewness and < 7.00 for kurtosis. Items PCW4, PCW2, and RH3 had the highest kurtosis (5.04, 5.53, 6.59, respectively).

#### Factorial validity and reliability

**Fig. 2:**
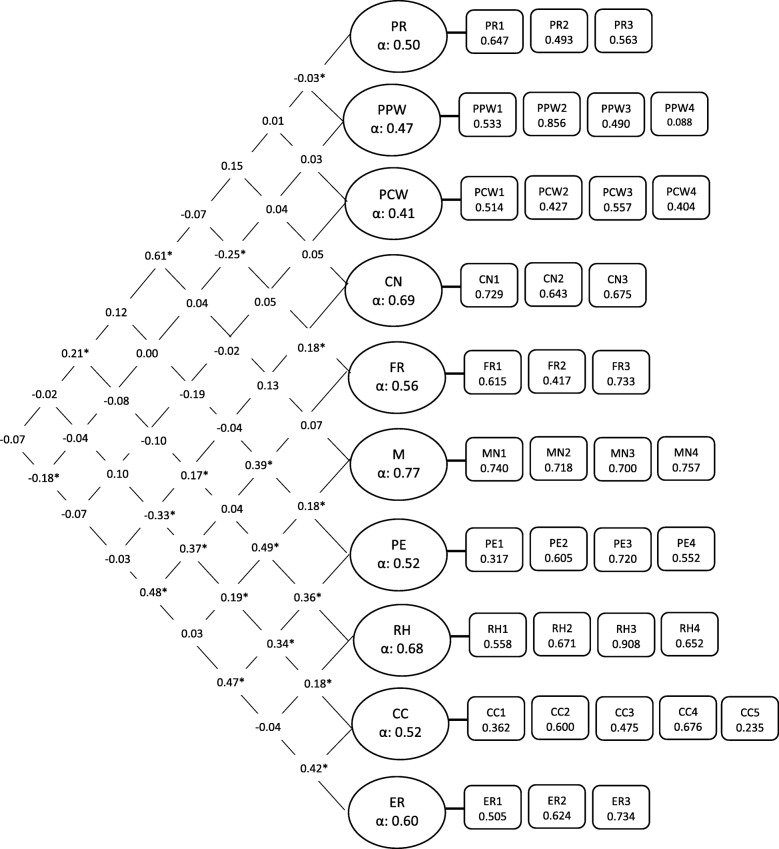
The Parental Child Feeding Practices Model in Study Two. The PCFP model with standardized estimates fitted in a sample of Study 2, factor correlations and reliabilities (*n* = 238)

We followed accepted methods for testing factorial validity using confirmatory factor analysis. Models were fitted with factor–factor correlation, uncorrelated errors, and variance on the first item fixed to one [35, 40]. The overall fit of the model was satisfactory based on accepted fit indices (see Methods). The χ2/df = 1.22 (*p* < .01); RMSEA = 0.030 (90% CI = 0.022–0.038); CFI = 0.915 and TLI = 0.903; with a weighted root mean square residual close to 1 (0.94). All of the factor-item loadings were above 0.4, except PPW4 (0.088) on perceived parent weight (PPW), PE1 (0.317) on pressure to eat (PE), and CC1 (0.362) on child control (CC) (see Fig. 2).

We sought discriminant validity of factor–factor correlations (Fig. 2). Overall, none of the factor–factor correlations were high enough (*r* = > 0.8) to show any overlap of factors [40]. Perceived responsibility (PR) was positively related to monitoring (*r* = 0.61, *p* < .05) and restriction for health (*r* = 0.21, *p* < .05). Concern about child weight was positively related to FR (*r* = 0.18, *p* < .05) and RH (*r* = 0.17, *p* < .05), and negatively related to child control (*r* = − 0.33, *p* < .05). Monitoring was positively related to PE (r = 0.18, *p* < .05), RH (*r* = 0.49, *p* < .05) and CC (*r* = 0.19, *p* < .05). Pressure to eat (PE) was positively related to RH (*r* = 0.36, *p* < .05), CC (*r* = 0.34, *p* < .05) and ER (*r* = 0.47, *p* < .05). Restriction for health (RH) was positively related to CC (*r* = 0.18, *p* < .05).

We tested the internal consistencies of the 10 subscales using Cronbach’s alpha. In general, the reliabilities were low, with only monitoring (M) showing acceptable value (0.77). Concern about child weight (CN) and restriction for health (RH) had reliability above 0.65. Two subscales with unacceptable internal consistency were perceived parent weight (PPW) (0.47) and perceived child weight (PCW) (0.41).

The convergent validity of the 10 subscales with nutrition status as three categories was tested using ANOVA (Table 3). Significant associations were found in two subscales, PCW and CN. Post hoc analysis of PCW shows significant differences between UW and NW (*p* < .05), UW and OW (*p* < .05) and NW and OW (*p* < .05). In the case of CN, post hoc analysis show a significant difference between UW and NW (*p* < .05).

**Table 3:** Convergent validity of PCFP subscales with nutritional status in Study 2

	Child nutritional status^c^			
	UW Mean (SD)	NW Mean (SD)	OW Mean (SD)	*p*-value
PR	3.88 (0.58)	3.87 (0.58)	3.90 (0.61)	0.98
PPW	3.03 (0.42)	3.09 (0.40)	3.26 (0.48)	0.06
PCW^a^	2.93 (0.34)	3.07 (0.27)	3.53 (0.42)	0.01^b^**
CN	2.67 (1.05)	3.12 (0.98)	2.93 (0.85)	0.01^b^**
FR	2.59 (0.74)	2.68 (0.76)	2.82 (0.63)	0.42
M	3.74 (0.74)	3.60 (0.83)	3.76 (0.63)	0.35
PE	3.81 (0.89)	3.74 (0.87)	3.99 (0.80)	0.51**
RH	4.13 (0.81)	4.01 (1.01)	4.16 (0.90)	0.83**
CC	3.05 (0.60)	2.88 (0.58)	3.11 (0.58)	0.06
ER	2.99 (0.72)	2.92 (0.81)	2.75 (0.64)	0.44

*UW* Underweight, *NW* Normal weight, *OW* Overweight

**p-value from Kruskal-Wallis test (mean score is not normally distributed)

^a^PCW 1–4

^b^significant mean difference (*p* < .05)

^c^Categories of child nutritional status according to the international (IOTF) childhood BMI cut-offs for overweight, obesity and thinness [31] were regrouped

## Discussion

This is the first study in a LMIC to validate measures of parenting child feeding practice. This included comprehensive adaptation of relevant subscales from existing measures to suit the context, and tests of their psychometric properties. Prior to this study, there had been, to our knowledge, only one study that tested the CFQ in a LMIC context, however they could not confirm the factorial structure of the CFQ in the setting [13]. In our research, the measures were tested using two sequential studies in different parts of Indonesia. The measures selected included both “coercive” and “structural” components of parent behaviors [14]. The subscales were reduced from 13 in Study 1 to ten in Study 2, based on statistical and substantive factors. Each of the 10 subscales and the instrument as a whole showed acceptable construct validity based on confirmatory factor analysis. There were, however, some issues related to low reliability, especially in the second study.

We overcame some known bias in previous validation studies that sampled highly educated parents [22], parents in higher [19] and lower [18] income groups, and the use of self-reported measures [16, 22]. Using samples from five sites in two provinces, our study collected data from a wide spectrum of respondents in rural, suburban, and urban settings. In general, children in our sample had higher levels of stunted growth and undernourishment than those in HIC, which allowed us to test the generalizability of the measures among a wider spectrum of children.

An important aspect of the study was that content validity was given attention in the selection of measures of child feeding practices in the setting. In addition, the study tested the content validity of the subscales and items by conducting a qualitative study with mothers during Study 2. A tested method used in cultural adaptation of measures from western countries was used to examine whether items that originated and had been tested elsewhere were appropriate to Indonesia [37]. All of the subscales (used as predetermined themes) were populated by text from the qualitative interviews, leading us to retain all 10 subscales and each of the items. The more recent conceptual distinction of between “coercive” and “structural” measures for PCFP was taken into consideration in the selection of measures to form the instrument tested.

An important finding of this study was that the original constructs for measuring control of feeding (pressure to eat, restriction, and monitoring) used in the development of the CFQ [9] were generalizable to a setting with double burden of malnutrition, with a significant proportion of children who were undernourished. There have been issues regarding the suitability of the PE items (PE1 in the German study and PE3 & PE4 in the study in Sweden) [16, 22]. In the only test of CFQ in a LMIC, Vietnam, the PE subscale was not validated. In Study 1, good factor loadings were found for the PE items, but the loading for PE1 was low in Study 2. Overall, the 10 subscales that were tested in this study showed acceptable psychometric properties [35]. The low scores on the measure for concern for child weight (CN), and specifically CN3 (how concerned are you about your child becoming overweight?), and our findings from the qualitative study seem to point to the fact that in general mothers in our sample did not consider the weight of the child to be an important consideration in their child feeding practice.

Our test of the convergent validity of the measures with BMI was not statistically significant, except for two subscales that measured mothers’ perception (PPW and PCW) and concern (CN). Other studies in HICs have also found insignificant relationship between child feeding practice subscales and children’s BMI [10, 41, 42]. In studies that found significant relationships between pressure to eat, restriction of food and BMI [9], the levels of overweight and obesity were higher. Significant correlation was seen only with pressure to eat in Hispanic and African-American groups [18]; however, the correlations were low (− 0.15 and − 0.16 respectively). Relative to the study by Kong et al. [18], our sample sizes were smaller. This may explain the lack of significance. In addition, pressure to eat in Indonesia may be differently applied to boys and girls, explaining the lower association with BMI. This requires further investigation.

There remain some limitations to our study. The findings cannot be generalized to the whole population of the country, as there are many different cultural groups in a diverse society such as Indonesia. While we used well-trained enumerators, we are cognizant of the limitations of interviews intended to elicit fine-grained distinctions about perceptions and behavior, especially when interviewees are less educated. We believe that social desirability bias, seen in other studies of parent feeding practices, may have been less prevalent among our respondents as in general there was not a high level of concern about the weight of the child. This was objectively seen in the concern for child weight scale (CN3), which had the highest flooring effect of all items. The Restriction for Weight scale in Study One was taken out to reduce respondent burden in Study Two. This limits the ability to measure any changes that take place over time where due to nutritional transition parents may impose restriction to child feeding. In addition, where useful researchers may need to consider adding this subscale for future cross sectional studies. The low reliabilities of some subscales are a cause for concern, compared to high reliabilities found in studies conducted in HIC. However, in Vietnam, the reliabilities for PE and PPW were even lower, 0.5 [13]. One possible explanation could be that the variation in education levels and low levels of education of mothers may have resulted in variations in their interpretation of the psychometric questions. There may be limitations in using measures for “structural” feeding practices developed in HIC, as local experts did not feel they were relevant to the context as they are very culture-specific. There is a need to develop scales on “structural” feeding practices from ground up, rather than adapting items from other contexts. We did not carry out further tests of convergent validity which is an area for future research.

## Conclusion

Most instruments that purport to measure parent child feeding practice have been developed in high-income countries where childhood obesity is a recognized problem. In many LMIC, there remains a double burden of undernutrition and obesity. Prior to this study, only one study had attempted to validate PCFP measures (specifically the CFQ) in a LMIC. This study fills this gap. Our study carried out tests of content validity to determine if the constructs and items were contextually relevant to the setting, and to test the measures’ factorial validity. The study provides evidence of acceptable factorial validity for the 10 subscales selected from well-tested instruments of PCFP (CFQ and CFPQ) for use in Indonesia. There are still areas that require further investigation.

## Additional files

Additional file 1: Associations between parental feeding subscales and correlation with child’s weight status. (DOCX 19 kb)

Additional file 2: a Characteristics of items and subscales used in Study One. b Characteristics of items and subscales used in Study Two. (DOCX 45 kb)

Additional file 3: Examples of texts from the interview transcripts. (DOCX 19 kb)

## Abbreviations

BMI: Body mass index
CC: Child control
CFA: Confirmatory factor analysis
CFI: Comparative Fit Index
CFPQ: Child feeding practice questionnaire
CFQ: Child Feeding Questionnaire
CHW: Community health worker
CN: Concern about child weight
EBV: Encourage balance and variety
Env: Environment
ER: Emotion regulation
FR: Food as reward
HIC: High-income countries
IOTF: International Obesity Task Force
ISP: Integrated Service Post
LMIC: Low and middle-income countries
M: Monitoring
PCFP: Parental child feeding practices
PCW: Perceived child weight
PE: Pressure to eat
PPW: Perceived parent weight
PR: Perceived responsibility
RH: Restriction for health
RMSEA: Root mean square error of approximation
RW: Restriction for weight
TLI: Tucker Lewis Index
WHO: World Health Organization
WLSMV: Weighted least square mean and variance adjusted
WRMR: Weighted root mean square residual
