# Supplementary material for: Validation of a measurement instrument for parental child feeding in a low and middle-income country
Source: Int J Behav Nutr Phys Act. 2018 Nov 20;15:113. doi: 10.1186/s12966-018-0736-7 (PMC6245694; doi:10.1186/s12966-018-0736-7)
Supplement: Supplementary file 1 — Associations between parental feeding subscales and correlation with child’s weight status. (DOCX 19 kb) [file 12966_2018_736_MOESM1_ESM.docx]

**Additional file 1. Associations between parental feeding subscales and correlation with child’s weight status**

**Associations between parental feeding subscales**

In the original study of Birch et al (2001) of the measures of parental perception, perceived responsibility (PR) was found to be positively associated with all of the controlled feeding practices, restriction (R), monitoring (MN) and pressure to eat (PE) [1]. These were confirmed in studies in Australia, Portugal, and Vietnam [2, 3, 4]. In a study in Sweden [5] a positive association was seen with R and PE. In a study among minority groups in the US [6] PR had a positive association only with MN, this was also seen in another Australian study [7]. PR was found to have low negative association with PE (r= -0.15) only in the Birch (2001) study [1].

Concern for child weight (CN) has mostly been positively associated only with restriction (R) [1, 3, 6, 7]. In Sweden, Nowicka et al. (2014) found positive association with PE and a negative association with MN [5]. The positive association with PE was also found among minority groups in the US [6].

Perception of child weigh (PCW) has been found to be negatively associated with pressure to eat in most studies, with a range from r= -0.11 [4] to -0.42 [3]. In contrast, CN had a strong positive relationship (r =0.48 and r =0.44) with restriction in African – American and Hispanic groups respectively in the US [6]. Three studies [1, 4, 5] also found a positive association of PCW with restriction (R).

**Factor correlations with child’s weight status**

Many studies have tested the hypothesis for the relationship of parental perceptions and child feeding with child weight following Birch (2001) who found that measures of parental perceptions were positively correlated and that PE was negatively related (r= -0.26) with child weight (weight for height) [1]. In other studies in the US, Anderson (2005) found the PCW was positively related to child’s BMI (r=0.38) and (r=0.42) in blacks and Hispanics respectively, but CN was only related among blacks (r=0.27) [8]. The same result that CN was related to child’s BMI among African Americans (AA) and a low negative association was found with PE (r=0.16) in the AA group in another study [6]. An Australian study found high positive correlation of child’s BMI with PCW and CN, and a negative correlation with PE [7]. In Sweden PE was negatively and R positively related to child BMI [5]. Real (2014) analysed BMI as a categorical variable and found similar results to Birch (2001) with PCW, CN and PE [1, 3]. In Vietnam the child’s BMI showed a low negative correlations with PE (r=-0.12 in urban and r=-0.05 in rural) and MN (r=-0.08 in urban and r=-0.09 in rural) [4].

**References:**

1. Birch LL, Fisher JO, Grimm-Thomas K, Markey CN, Sawyer R, Johnson SL. Confirmatory factor analysis of the Child Feeding Questionnaire: a measure of parental attitudes, beliefs and practices about child feeding and obesity proneness. Appetite. 2001; doi:10.1006/appe.2001.0398.
2. Liu W, Mallan KM, Mihrshahi S, Daniels LA. Feeding beliefs and practices of Chinese immigrant mothers: Validation of a modified version of the Child Feeding Questionnaire. Appetite. 2014; doi:10.1016/j.appet.2014.04.030.
3. Real H, Oliveira A, Severo M, Moreira P, Lopes C. Combination and adaptation of two tools to assess parental feeding practices in pre-school children. Eating Behaviors. 2014; doi: 10.1016/j.eatbeh.2014.04.009 .
4. Do LM, Eriksson B, Tran TK, Petzold M, Ascher H. Feeding of preschool children in Vietnam: a study of parents’ practices and associated factors. BMC Nutrition. 2015; doi 10.1186/s40795-015-0011-0.
5. Nowicka P, Sorjonen K, Pietrobelli A, Flodmark K, Faith MS. Parental feeding practices and associations with child weight status: Swedish validation of the Child Feeding Questionnaire finds parents of 4-year-olds less restrictive. Appetite. 2014*;* doi:10.1016/j.appet.2014.06.027.
6. Kong A, Vijayasiri G, Fitzgibbon ML, Schiffer LA, Campbell RT. Confirmatory factor analysis and measurement invariance of the Child Feeding Questionnaire in low-income Hispanic and African-American mothers with preschool-age children. Appetite. 2015; doi:10.1016/j.appet.2015.02.027.
7. Corsini N, Danthiir V, Kettler L, Wilson C. Factor structure and psychometric properties of the Child Feeding Questionnaire in Australian preschool children. Appetite. 2008; doi: 10.1016/j.appet.2008.02.013
8. Anderson CB, Hughes SO, Fisher JO, Nicklas TA. Cross-cultural equivalence of feeding beliefs and practices: the psychometric properties of the child feeding questionnaire among Blacks and Hispanics. Preventive Medicine. 2005; doi:10.1016/j.ypmed.2005.01.003.
