# Supplementary material for: Validation of a measurement instrument for parental child feeding in a low and middle-income country
Source: Int J Behav Nutr Phys Act. 2018 Nov 20;15:113. doi: 10.1186/s12966-018-0736-7 (PMC6245694; doi:10.1186/s12966-018-0736-7)
Supplement: Supplementary file 2 — a Characteristics of items and subscales used in Study One. b Characteristics of items and subscales used in Study Two. (DOCX 45 kb) [file 12966_2018_736_MOESM2_ESM.docx]

**Additional file 2a. Characteristics of items and subscales used in Study One**

| **Items by subscales** | | **Mean** | **Std. Dev** | **Skewness** | **Kurtosis** | **Average inter-item correlation** |
| --- | --- | --- | --- | --- | --- | --- |
| Perceived Responsibility^a^ | |  |  |  |  |  |
| PR1 | When your child is at home, how often are you responsible for feeding him or her? | 4.43 | 0.93 | -1.49 | 4.44 | 0.17 |
| PR2 | How often are you responsible for deciding what your child’s portion sizes are? | 4.01 | 1.43 | -1.18 | 2.92 | 0.23 |
| PR3 | How often are you responsible for deciding if your child has eaten the right kind of foods? | 4.16 | 1.10 | -1.12 | 3.30 | 0.46 |
|  |  |  |  |  |  |  |
| Perceived Parental Weight^a^ | |  |  |  |  |  |
| PPW1 | Your childhood (5 – 10 years old) | 2.78 | 0.65 | 0.39 | 2.93 | 0.36 |
| PPW2 | Your adolescence | 2.90 | 0.70 | 0.25 | 2.42 | 0.30 |
| PPW3 | Your 20s | 2.90 | 0.73 | 0.25 | 2.23 | 0.27 |
| PPW4 | At present | 3.21 | 0.78 | -0.31 | 1.86 | 0.57 |
|  |  |  |  |  |  |  |
| Perceived Child Weight^a^ | |  |  |  |  | 0.31* |
| PCW1 | Your child during the first year of life | 2.90 | 0.69 | -0.11 | 2.68 |  |
| PCW2 | Your child as a toddler | 2.44 | 0.58 | 0.53 | 2.70 |  |
| PCW3 | Your child as a pre-schooler** |  |  |  |  |  |
|  |  |  |  |  |  |  |
| Child Control^b^ | |  |  |  |  |  |
| CC1 | Do you let your child eat whatever s/he wants? | 3.36 | 1.26 | -0.26 | 2.30 | 0.23 |
| CC2 | At dinner, do you let this child choose the foods s/he wants from what is served? | 4.14 | 1.22 | -1.32 | 3.81 | 0.19 |
| CC3 | If this child does not like what is being served, do you make something else? | 3.47 | 1.49 | -0.48 | 1.85 | 0.27 |
| CC4 | Do you allow this child to eat snacks whenever s/he wants? | 3.63 | 1.26 | -0.51 | 2.33 | 0.19 |
| CC5 | Do you allow this child to leave the table when s/he is full, even if your family is not done eating? | 3.49 | 1.86 | -0.51 | 1.35 | 0.23 |
|  |  |  |  |  |  |  |
| Concern about Child Weight^a^ | |  |  |  |  | 0.24*** |
| CN1 | How concerned are you about your child eating too much when you are not around her? | 3.05 | 1.71 | -0.06 | 1.34 |  |
| CN2 | How concerned are you about your child having to diet to maintain a desirable weight? | 2.73 | 1.66 | 0.21 | 1.42 |  |
| CN3 | How concerned are you about your child becoming overweight? | 1.51 | 1.17 | 2.13 | 6.07 |  |
| Emotion Regulation^b^ | |  |  |  |  |  |
| ER1 | When this child gets fussy, is giving him/her something to eat or drink the first thing you do? | 3.38 | 1.51 | -0.37 | 1.76 | 0.85 |
| ER2 | Do you give this child something to eat or drink if s/he is bored even if you think s/he is not hungry? | 3.03 | 1.56 | -0.11 | 1.52 | 0.62 |
| ER3 | Do you give this child something to eat or drink if s/he is upset even if you think s/he is not hungry? | 2.96 | 1.60 | -0.02 | 1.45 | 0.61 |
|  |  |  |  |  |  |  |
| Food as Reward^b^ | |  |  |  |  |  |
| FR1 | I offer sweets (candy, ice-cream, cake, pastries) to my child as a reward for good behaviour. | 2.70 | 1.85 | 0.30 | 1.22 | 0.54 |
| FR2 | I withhold sweets/dessert from my child in response to bad behaviour. | 2.92 | 1.79 | 0.05 | 1.21 | 0.60 |
| FR3 | I offer my child his/her favourite foods in exchange for good behaviour. | 3.53 | 1.64 | -0.60 | 1.72 | 0.57 |
|  |  |  |  |  |  |  |
| Monitoring^b^ | |  |  |  |  |  |
| M1 | How much do you keep track of the sweets (candy, ice-cream, cake, pies, pastries) that your child eats? | 4.27 | 1.26 | -1.73 | 4.72 | 0.67 |
| M2 | How much do you keep track of the snack food (potato chips, Doritos, cheese puffs) that your child eats? | 4.41 | 1.08 | -2.07 | 6.55 | 0.63 |
| M3 | How much do you keep track of the high-fat foods that your child eats? | 4.19 | 1.22 | -1.49 | 4.21 | 0.67 |
| M4 | How much do you keep track of the sugary drinks (soda/pop, kool-aid) this child drinks? | 4.27 | 1.25 | -1.75 | 4.86 | 0.65 |
|  |  |  |  |  |  |  |
| Pressure to Eat^b^ | |  |  |  |  |  |
| PE1 | My child should always eat all of the food on his/her plate. | 4.23 | 1.28 | -1.55 | 4.15 | 0.41 |
| PE2 | If my child says, ‘‘I’m not hungry,’’ I try to get him/her to eat anyway. | 3.72 | 1.64 | -0.82 | 1.97 | 0.34 |
| PE3 | If my child eats only a small helping, I try to get him/her to eat more. | 3.86 | 1.53 | -1.00 | 2.40 | 0.33 |
| PE4 | When he/she says he/she is finished eating, I try to get my child to eat one more (two more, etc.) bites of food. | 3.94 | 1.50 | -1.12 | 2.69 | 0.38 |
|  |  |  |  |  |  |  |
| Restriction for Health^b^ | |  |  |  |  |  |
| RH1 | If I did not guide or regulate my child’s eating, s/he would eat too much of his/her favourite foods. | 4.35 | 1.29 | -1.84 | 4.93 | 0.46 |
| RH2 | If I did not guide or regulate my child’s eating, he/she would eat too many junk foods. | 4.28 | 1.33 | -1.65 | 4.25 | 0.37 |
| RH3 | I have to be sure that my child does not eat too much of his/her favourite foods. | 4.27 | 1.21 | -1.61 | 4.49 | 0.43 |
| RH4 | I have to be sure that my child does not eat too many sweets (candy, ice-cream, cake or pastries). | 4.34 | 1.31 | -1.81 | 4.70 | 0.54 |
|  |  |  |  |  |  |  |
| Restriction for Weight^b^ | |  |  |  |  |  |
| RW1 | I have to be sure that my child does not eat too many high-fat foods. | 3.64 | 1.69 | -0.71 | 1.75 | 0.46 |
| RW2 | I encourage my child to eat less so he/she won’t get fat. | 1.32 | 0.97 | 3.03 | 10.92 | 0.41 |
| RW3 | I give my child small helpings at meals to control his/her weight. | 1.27 | 0.87 | 3.33 | 13.27 | 0.39 |
| RW4 | If my child eats more than usual at one meal, I try to restrict his/her eating at the next meal. | 1.55 | 1.27 | 2.12 | 5.83 | 0.39 |
| RW5 | I restrict the food my child eats that might make him/her fat. | 1.42 | 1.16 | 2.54 | 7.70 | 0.36 |
| RW6 | There are certain foods my child shouldn’t eat because they will make him/her fat. | 1.40 | 1.13 | 2.61 | 8.10 | 0.37 |
| RW7 | I don’t allow my child to eat between meals because I don’t want him/her to get fat. | 1.34 | 1.00 | 2.93 | 10.25 | 0.38 |
| RW8 | I often put my child on a diet to control his/her weight. | 2.62 | 1.88 | 0.38 | 1.23 | 0.44 |

^a^Subscales and items were taken from Birch et al. (2001)

^b^Subscales and items were taken from Musher-Eizenman et al. (2007)

*Average inter-item correlation for PCW1 and PCW2, PCW3 was omitted in CFA

**PCW3 has 111 missing values (this item was not applicable to some children in the targeted age group in this study as they were not yet in preschool age group), PCW3 was omitted in CFA

***Average inter-item correlation for CN1 and CN2, CN3 was omitted in CFA

**Additional file 2b. Characteristics of items and subscales used in Study Two**

| **Items by subscales** | | **Mean** | **Std. Dev** | **Skewness** | **Kurtosis** | **Average inter-item correlation** |
| --- | --- | --- | --- | --- | --- | --- |
| Perceived Responsibility^a^ | |  |  |  |  |  |
| PR1 | When your child is at home, how often are you responsible for feeding him or her? | 4.26 | 0.65 | -0.59 | 3.52 | 0.20 |
| PR2 | How often are you responsible for deciding what your child’s portion sizes are? | 3.69 | 1.02 | -0.63 | 2.99 | 0.30 |
| PR3 | How often are you responsible for deciding if your child has eaten the right kind of foods? | 3.68 | 0.77 | -0.28 | 3.09 | 0.25 |
|  |  |  |  |  |  |  |
| Perceived Parental Weight^a^ | |  |  |  |  |  |
| PPW1 | Your childhood (5 – 10 years old) | 2.87 | 0.61 | 0.07 | 4.15 | 0.16 |
| PPW2 | Your adolescence | 2.96 | 0.67 | 0.05 | 3.28 | 0.12 |
| PPW3 | Your 20s | 3.02 | 0.65 | -0.02 | 3.57 | 0.15 |
| PPW4 | At present | 3.47 | 0.77 | -0.08 | 2.62 | 0.31 |
|  |  |  |  |  |  |  |
| Perceived Child Weight* | |  |  |  |  |  |
| PCW1 | Your child at present | 2.87 | 0.54 | -0.09 | 4.34 | 0.14 |
| PCW2 | Your child at birth | 3.12 | 0.55 | 0.52 | 5.53 | 0.16 |
| PCW3 | Your child at 6 month | 3.29 | 0.68 | 0.35 | 3.60 | 0.13 |
| PCW4 | Your child at 1 year old | 2.95 | 0.55 | 0.12 | 5.04 | 0.16 |
| PCW5 | Your child at 2 years old** |  |  |  |  |  |
|  |  |  |  |  |  |  |
| Concern about Child Weight^a^ | |  |  |  |  |  |
| CN1 | How concerned are you about your child eating too much when you are not around her? | 2.98 | 1.16 | -0.02 | 2.22 | 0.41 |
| CN2 | How concerned are you about your child having to diet to maintain a desirable weight? | 2.98 | 1.34 | 0.04 | 1.87 | 0.44 |
| CN3 | How concerned are you about your child becoming overweight? | 2.84 | 1.38 | 0.11 | 1.73 | 0.41 |
|  |  |  |  |  |  |  |
| Food as Reward^b^ | |  |  |  |  |  |
| FR1 | I offer sweets (candy, ice-cream, cake, pastries) to my child as a reward for good behaviour. | 2.69 | 0.99 | -0.05 | 2.72 | 0.29 |
| FR2 | I withhold sweets/dessert from my child in response to bad behaviour. | 2.07 | 1.11 | 0.72 | 2.65 | 0.39 |
| FR3 | I offer my child his/her favourite foods in exchange for good behaviour. | 3.22 | 0.97 | -0.17 | 2.93 | 0.21 |
|  |  |  |  |  |  |  |
| Monitoring^b^ | |  |  |  |  |  |
| M1 | How much do you keep track of the sweets (candy, ice-cream, cake, pies, pastries) that your child eats? | 3.74 | 0.91 | -0.48 | 2.95 | 0.48 |
| M2 | How much do you keep track of the snack food (potato chips, Doritos, cheese puffs) that your child eats? | 3.68 | 1.01 | -0.52 | 2.74 | 0.43 |
| M3 | How much do you keep track of the high-fat foods that your child eats? | 3.44 | 0.99 | -0.29 | 2.65 | 0.46 |
| M4 | How much do you keep track of the sugary drinks (soda/pop, kool-aid) this child drinks? | 3.82 | 1.14 | -0.92 | 3.08 | 0.46 |
|  |  |  |  |  |  |  |
| Pressure to Eat^b^ | |  |  |  |  |  |
| PE1 | My child should always eat all of the food on his/her plate. | 4.05 | -1.24 | -1.03 | 2.89 | 0.29 |
| PE2 | If my child says, ‘‘I’m not hungry,’’ I try to get him/her to eat anyway. | 3.49 | 1.54 | -0.57 | 1.86 | 0.19 |
| PE3 | If my child eats only a small helping, I try to get him/her to eat more. | 4.02 | 1.24 | -1.20 | 3.40 | 0.14 |
| PE4 | When he/she says he/she is finished eating, I try to get my child to eat one more (two more, etc.) bites of food. | 3.59 | 1.42 | -0.71 | 2.20 | 0.23 |
|  |  |  |  |  |  |  |
| Restriction for Health^b^ | |  |  |  |  |  |
| RH1 | If I did not guide or regulate my child’s eating, s/he would eat too much of his/her favourite foods. | 3.66 | 1.40 | -0.72 | 2.25 | 0.39 |
| RH2 | If I did not guide or regulate my child’s eating, he/she would eat too many junk foods. | 3.87 | 1.60 | -1.00 | 2.28 | 0.33 |
| RH3 | I have to be sure that my child does not eat too much of his/her favourite foods. | 4.49 | 1.09 | -2.17 | 6.59 | 0.31 |
| RH4 | I have to be sure that my child does not eat too many sweets (candy, ice-cream, cake or pastries). | 4.26 | 1.11 | -1.51 | 4.53 | 0.34 |
|  |  |  |  |  |  |  |
| Child Control^b^ | |  |  |  |  |  |
| CC1 | Do you let your child eat whatever s/he wants? | 2.76 | 0.98 | 0.00 | 3.04 | 0.18 |
| CC2 | At dinner, do you let this child choose the foods s/he wants from what is served? | 3.05 | 1.07 | -0.01 | 2.75 | 0.14 |
| CC3 | If this child does not like what is being served, do you make something else? | 3.14 | 1.02 | -0.04 | 2.96 | 0.21 |
| CC4 | Do you allow this child to eat snacks whenever s/he wants? | 3.20 | 0.95 | 0.15 | 2.81 | 0.13 |
| CC5 | Do you allow this child to leave the table when s/he is full, even if your family is not done eating? | 2.65 | 1.06 | -0.08 | 2.40 | 0.24 |
|  |  |  |  |  |  |  |
| Emotion Regulation^b^ | |  |  |  |  |  |
| ER1 | When this child gets fussy, is giving him/her something to eat or drink the first thing you do? | 3.51 | 1.06 | -0.36 | 2.67 | 0.43 |
| ER2 | Do you give this child something to eat or drink if s/he is bored even if you think s/he is not hungry? | 2.69 | 1.00 | 0.08 | 2.87 | 0.27 |
| ER3 | Do you give this child something to eat or drink if s/he is upset even if you think s/he is not hungry? | 2.60 | 1.03 | 0.00 | 2.39 | 0.29 |

^a^Subscales and items were taken from Birch et al. (2001)

^b^Subscales and items were taken from Musher-Eizenman et al. (2007)

*Modified subscale and items of PCW

**PCW5 was omitted in the CFA
