# Supplementary material for: Validation of a measurement instrument for parental child feeding in a low and middle-income country
Source: Int J Behav Nutr Phys Act. 2018 Nov 20;15:113. doi: 10.1186/s12966-018-0736-7 (PMC6245694; doi:10.1186/s12966-018-0736-7)
Supplement: Supplementary file 3 — Examples of texts from the interview transcripts. (DOCX 19 kb) [file 12966_2018_736_MOESM3_ESM.docx]

**Additional file 3. Examples of texts from the interview transcripts**

| **Subscale** | **Item** | **Example of quotation for each item from the transcripts** |
| --- | --- | --- |
| **Perceived Responsibility** | When your child is at home, how often are you responsible for feeding him or her? | *Well, I should provide food and give it to them everyday, but I do not set up three times meal times. My younger daughter sometimes eats together with her (older) sister. My first daughter can choose foods from the table, then I just take food for my younger daughter in particular portion. Sometimes I take the food for both of them and feed them together at home. (R3)*  *I am happy to prepare foods for my kid every day. Usually, he says “Mom, please cook soup for me..”, Or “Mom, tomorrow, please fry an egg,” I said “Yes.” It depends on what my kid wants. I usually ask him "what food you want me to cook tomorrow?" If I feel tired, I buy some side dishes, cooked side dishes. I don’t like packaged food. (15)* |
|  | How often are you responsible for deciding what your child’s portion sizes are? | *Usually, I watch them, how much they take the rice and I see the rest, compared to the full portion and check it, and evaluate it. Based from this, I can assume that's enough. (R1)*  *I should control what she eats and its amount. I want to make sure she eats healthy food in appropriate portion for her age. That is my responsibility Miss. (R16)* |
|  | How often are you responsible for deciding if your child has eaten the right kind of foods? | *I try to provide various foods so he would not get bored. As I told you earlier, for me healthy food is the fresh one. Everytime I buy food, it should be the good one, fresh. For example, if I want to buy spinach, I would select spinach, which has perforated leaves. It means that vegetables were not sprayed with pesticides. The caterpillar would not eat the leaves that were sprayed with pesticides by the farmer, right? The same reason also applies for other vegetables, such as broccoli. I have to make sure that my son eats the right foods every day (smile). (R11)*  *I am not sure which foods are better, but I think the right foods should be varied and that is the important thing. Every day, we provide different food, Miss, should be like that. There is a variation in the menu. At least we always change the vegetable menu. We cook similar animal-based food every day such as chicken and egg. We also provide tofu and tempeh. As for vegetables, we use different fresh vegetables. (R12)* |
| **Perceived Parental Weight** | Your childhood (5 – 10 years) | *I have been overweight since my childhood; since I was a little girl. I was never thin. (R16)*  *I was so skinny when I was a child. (R21)* |
|  | Your adolescence | *I just lazy and I did not eat enough foods at that time. When I was in junior high school, I was only thirty-three kilos. So thin. (R7)*  *I was getting fat when I was fifteen. (R23)* |
|  | Your 20s | *I was forty-two kilos in my 20s, before having kids. (R2)*  *I was so small when I was young...Yes.. my heaviest scale was only forty two, only forty two.. Forty two kilos (R9)* |
|  | At present | *I am overweight. Yesterday I put my jeans on, then I realized it did not fit me. I am fat. I can’t move freely. I wear my old jeans, and I can’t move freely. I prefer being skinny...I feel so heavy when I am walking and working. (R10)*  *Well, you can see that I am quite big and fat now. Well, I feel better when I had a healthy weight. I think it was easier for me to move when I had normal weight. I feel uneasy when I am fat. (R13)* |
| **Pressure to Eat** | My child should always eat all of the food on his/her plate. | *If I take her to walk, she can eat a lot and finish the foods on the plate. So basically, I have to stop all of my work and take her to eat because she tends to stop anytime and not finish her meals. She has to finish her meals. (R8)*  *Yes, he should eat all foods in the plate. I’ll follow him and bring his meals wherever he wants to go, but sometimes just around the house. (R10)* |
|  | If my child says, ‘‘I’m not hungry,’’ I try to get him/her to eat anyway. | *Yes, even though she said 'not hungry,' I would feed her. I gave her something, like toys. When she was playing, I gave her three or four spoons. (R6)*  *Even my child doesn't want to eat, and she says that she is not hungry; I try to get her to eat. I need to take her and walk around the house and give the food at the same time. (R16)* |
|  | If my child eats only a small helping, I try to get him/her to eat more | *Hmm I try to give more food to her, and she asks me to ride her a bicycle around our house. She only eats small portion of her foods. (R2)*  *Yes I try to get him to eat more. I take him to watch the train and feed him. (R14)* |
|  | When he/she says he/she is finished eating, I try to get my child to eat one more (two more, etc.) bites of food. | *When he is finished with his foods, I usually ask him: "Do you want more?" He says no, but I still try to give him one bite. He smiles but run because he does not want to eat anymore (laughing). (R15)*  *My daughter can tell if she feels full. Even though she is full, I always try to give another spoon. She usually can eat one or two spoons, but after that, she will say "Enough, Mom." (R1)* |
